# Nanoelectrode Arrays Fabricated by Thermal Nanoimprint Lithography for Biosensing Application

**DOI:** 10.3390/bios10080090

**Published:** 2020-08-05

**Authors:** Alessandra Zanut, Alessandro Cian, Nicola Cefarin, Alessandro Pozzato, Massimo Tormen

**Affiliations:** 1 Department of Physics, University of Trieste, P.le Europa 1, 34100 Trieste, Italy; cefarin@iom.cnr.it; 2 IOM-CNR, TASC Laboratory, Area Science Park—Basovizza, S.S 14 Km 163.5, I-34149 Trieste, Italy; massimo.tormen@thundernil.com; 3 ThunderNIL srl, via Foscolo 8, I-35131 Padova, Italy; acian@fbk.eu (A.C.); alessandro.pozzato@thundernil.com (A.P.); 4 Center for Materials and Microsystems, Fondazione Bruno Kessler, 38123 Trento, Italy

**Keywords:** nanoelectrode arrays, nanoimprint lithography, biosensor, electrochemistry

## Abstract

Electrochemical sensors are devices capable of detecting molecules and biomolecules in solutions and determining the concentration through direct electrical measurements. These systems can be miniaturized to a size less than 1 µm through the creation of small-size arrays of nanoelectrodes (NEA), offering advantages in terms of increased sensitivity and compactness. In this work, we present the fabrication of an electrochemical platform based on an array of nanoelectrodes (NEA) and its possible use for the detection of antigens of interest. NEAs were fabricated by forming arrays of nanoholes on a thin film of polycarbonate (PC) deposited on boron-doped diamond (BDD) macroelectrodes by thermal nanoimprint lithography (TNIL), which demonstrated to be a highly reliable and reproducible process. As proof of principle, gliadin protein fragments were physisorbed on the polycarbonate surface of NEAs and detected by immuno-indirect assay using a secondary antibody labelled with horseradish peroxidase (HRP). This method allows a successful detection of gliadin, in the range of concentration of 0.5–10 μg/mL, by cyclic voltammetry taking advantage from the properties of NEAs to strongly suppress the capacitive background signal. We demonstrate that the characteristics of the TNIL technology in the fabrication of high-resolution nanostructures together with their low-cost production, may allow to scale up the production of NEAs-based electrochemical sensing platform to monitor biochemical molecules for both food and biomedical applications.

## 1. Introduction

Chasing an ever-increasing need for sensing elements that are emerging in different fields and applications, electrochemical biosensors are essential pillars in future scenarios. These sensors play a significant role in biomedical and environmental monitoring also for their exceptional attributes, such as being easy-to-operate, economical, sensitive, and portable [1,2,3].

Electrochemical sensors are devices capable of detecting molecules and biomolecules in solutions and determining the concentration through direct electrical measurements, arising from the change in the redox state of the analyte, monitored through classical electroanalytical techniques [4,5].

Remarkably, recent achievements in nanoscience and nanotechnology have demonstrated the potential for improving greatly both the sensitivity and selectivity of electrochemical sensors and biosensors [6,7]. In fact, an electrochemical sensor can be miniaturized to a size less than 1 µm or to small-size arrays of nanoelectrodes (NEA), offering advantages in terms of increased sensitivity and compactness [8,9].

A NEA is made by a very large number of very small nanoelectrodes separated by an electrical insulator, with density between 10^6^ and 10^8^ electrodes/cm^2^. When the electrode’s critical dimension becomes comparable to the electrical double layer thickness or to the molecular size, the experimental behavior starts to deviate from extrapolations of behavior of larger electrodes [10,11].

Indeed, the electrochemical characteristics that distinguish nanoelectrodes arrays from conventional macro-(mm-sized) or even microelectrodes (μm-sized) are (i) the dramatic enhancement of diffusive mass fluxes to the nanoelectrode, (ii) the lower double-layer charging (capacitive) currents, and (iii) an extreme sensitivity to the kinetics of the charge transfer process [12,13,14,15]. All these factors contribute to a significant improvement of detection limits [16,17,18].

Moreover, unlike individual nanoelectrodes, NEA provide higher faradaic currents, thus require low amplification and avoid the use of shielding devices such as a Faraday cage [19,20].

In fact, NEA can exhibit different diffusion regimes that can be divided into five categories [15]: (i) planar diffusion over each microelectrode, (ii) mixed diffusion over each microelectrode (transition between planar and hemispherical diffusion), (iii) diffusion over each hemispherical microelectrode, (iv) diffusion mixed with onset of overlap of diffusion layers, and (v) planar diffusion over the entire array. The characteristics of the time-dependent diffusion profile of electrode arrays depend primarily on the relative interelectrode spacing, d/r (where d is the center-to-center distance between electrodes and r is the radius of nanoelectrode), as well as on the time-scale of the experiment [21].

In this context, methods capable of producing nanoelectrodes and of controlling their dimensions in a reproducible manner are of fundamental importance.

Over the past decades, the rapid development of top-down lithographic methods, such as ion beam lithography [22], electron beam lithography (EBL) [21], or scanning probe lithography [23,24], were extensively used to achieve high-resolution nanostructures with precise position and size down to a scale of a few nanometers. This spatial resolution capabilities have been indeed exploited to prepare ordered arrays of nanoelectrodes [8,25,26]. Among top-down lithographic methods, electron beam lithography (EBL) showed to be able to create synthetic surfaces with sub-50 nm spatial resolution and a well-defined control of the topographical features [8]. However, EBL process is expensive and time consuming making this technique non suitable for large-scale production of electrochemical nanostructured probes.

Herein, we present the fabrication of nanoelectrodes (NEA) on a polycarbonate (PC) resist film deposited on a boron-doped diamond (BDD) electrode through a thermal nanoimprinting (TNIL) process. TNIL has become very popular due to the low cost of its implementation (at least at an entry level) and high throughput for manufacturing in parallel patterns of nanostructures over large areas [27,28].In the imprint process, a mold with specific nanostructures on its surface is used to indent the same nanostructures in a thin thermoplastic film deposited on a substrate while the latter is in a melt state, i.e., during a thermomechanical cycle. This approach leads to the formation of recessed nanoelectrodes in the polymeric film that can be subsequently transferred to the substrate by plasma etching, after the removal of a residual resist that usually remains under the compressed areas [29,30].

The use of PC as resist was previously demonstrated by our group for the fabrication of NEAs by e-beam lithography on gold macroelectrodes [31] and BDD [8]. The most interesting properties of this polymer for nanofabrication purposes are the high lithographic contrast, which allows the creation of structures of dimensions less than 100 nm, and the chemical stability, which guarantees a long-term use in electrochemical solutions. The attractiveness of this polymer resides also in the possibility to functionalize its surface with biological molecules (DNA and proteins) enabling the creation of a biosensing platform. This is possible due to the natural presence of carboxylic groups on the PC surface that may interact with the amino groups present in different biomolecules. Several groups already used this strategy for detecting DNA [8,32,33] proteins [34,35], or enzyme substrates [36], exploiting nanoelectrode ensembles (NEE). NEE, however, present long and laborious manufacturing processes and do not allow to arbitrarily control the position and size of the nanoelectrodes.

**Figure 1:**
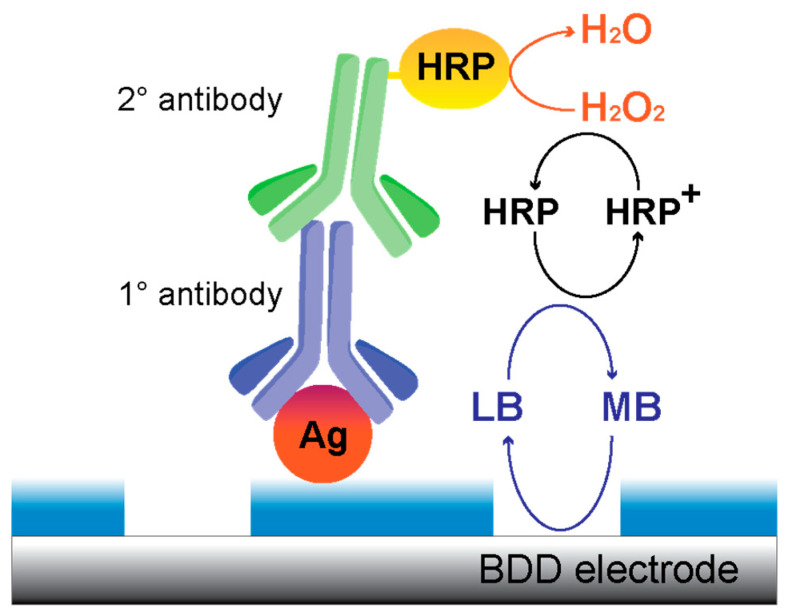
Schematic representation of the arrays of nanoelectrodes (NEA)-gliadin. The polycarbonate (PC) surface is exploited to immobilize the antigen (Ag) protein, gliadin. The assay is performed by molecular recognition of the target by the primary antibody anti-gliadin IgG and a subsequent secondary antibody anti-IgG labeled with horse radish peroxidase (HRP) enzyme. The electrocatalytic cycle was generated by adding the enzyme substrate H_2_O_2_ and the mediator methylene blue (MB).

In this work, we studied the possibility to use NEA fabricated by TNIL as electrochemical biosensing platform using cyclic voltammetry as transduction method. As testbed, we used the protein gliadin as antigen. Gliadin represents the soluble component of gluten, which is present in wheat and several other cereals, and is the main cause of the autoimmune reaction that occurs in coeliac patients. The only known method to treat coeliac disease to date is a lifelong gluten-free diet [37]; thus, the reliable labeling of gluten-free products is essential, and the detection of gliadin is of great importance in the control of gluten free foods. Patients with celiac disease should limit their daily gluten intake to no more than 10–50 mg. Most health authorities define gluten-free products as containing less than 20 parts per million gluten [38]. Gliadin was physisorbed on the PC film surface and detected by a monoclonal primary antibody specific for gliadin, followed by the binding of a secondary antibody labelled with horseradish peroxidase (HRP) (Figure 1). Finally, the binding of HRP is detected by adding H_2_O_2_ as substrate and methylene blue (MB) as redox mediator, thus modulating, via its electrochemical reduction, the enzymatic reduction of H_2_O_2_ by HRP.

Herein, we present a characterization of the NEA platform and the optimization of its analytical performances.

## 2. Materials and Methods

### 2.1. Materials and Instruments

Si ‹100› substrates coated with a 400 nm thick layer of BDD (Adamant Technologies SA, La Chaux de Fonds, Switzerland) were used as conductive layer for NEAs fabrication.

Polycarbonate solution (4% w/v) was obtained by dissolution of solid polycarbonate Makrolon (Bayer Sheet Europe, Darmstadt, Germany) in cyclopentanone. Methylene blue solution (0.01 mM) was prepared by solubilizing methylene blue powder, purchased from Acef Spa (Piacenza, Italy)., in PBS. Recombinant Wheat Gliadin protein (19 kDa), mouse monoclonal antigliadin antibody, and horseradish peroxidase (HRP)-labelled goat antimouse IgG secondary antibody were purchased from Abcam^®^ (Cambridge, UK). Other chemicals were purchased from Sigma-Aldrich (St. Luis, MO, USA), unless otherwise stated, and were used as received. Purified water was obtained by a Milli-RO plus Milli-Q (Millipore, Burlington, VT, USA) system.

Electrochemical measurements were carried out at room temperature in a three-electrode cell with a platinum coil counter electrode and an Ag/AgCl (KCl saturated) reference electrode, connected to a CHI Model 660B potentiostat.

Scanning electron microscopy (SEM) of the NEA was performed using a ZEISS, SUPRA 40-25-40 Carl Zeiss SMT Ltd. System (Carl Zeiss Microscopy, New York, NY, USA). 

### 2.2. Stamp Fabrication

For the nanoimprint lithography process, we used a stamp with a nanopillars array obtained by replicating a commercial silicon master consisting of a square array of nanoholes. The polycarbonate film was patterned by indenting the pillars in the PC, i.e., forcing the displacement of the polymer from the area of the pillars on the stamp during the NIL thermomechanical cycle.

The master for NEAs fabrication by TNIL was obtained according to the following procedure. First, a Si ‹100› substrate was spin coated with mr-I 7010E (Micro resist technology GmbH, Berlin, Germany) at 2000 rpm, which was annealed at 140 °C for 2 min. A film of 115 nm was obtained using these parameters. Then, a commercial master (AMO GmbH, Aachen, Germany), consisting of holes of 800 nm period, 400 nm diameter, and a depth of 400 nm, was replicated by TNIL process applying 10 MPa pressure for 15 min at 100 °C using press with heating plates. After that, a reactive ion etching in an inductive coupled plasma (ICP) reactor was used to transfer the pillars pattern on silicon. The process consists of a 15 s O_2_ plasma step (at 200 W and 10 W for coil and platen power with 40 sccm oxygen flow) to remove residual layer, followed by a fluorine-based plasma (at 400 and 20 W for coil and platen power, respectively, with SF_6_/C_4_F_8_/Ar gas mixture at 30/60/10 sccm flow), for silicon etching, and a final plasma ashing step (at 800 W and 20 W for coil and platen power with 50 sccm oxygen flow) to remove the resist mask. Table 1 gives the full list of the RIE-ICP parameters selected for the fabrication. Prior to use, the stamp was functionalized in vapor phase, with a monolayer of octyl-trichlorosilane for easy release, according to common procedures.

### 2.3. NEA Fabrication

Arrays of nanoelectrodes were fabricated by TNIL. Polycarbonate was spin-coated at 2000 rpm on a 400 nm thick layer of boron-doped diamond CVD deposited on heavily p-doped silicon. Polycarbonate films were annealed for 30 min at 180 °C to remove residual solvent. The average film thickness of PC films prepared from solutions at 4% in cyclopentanone measured by profilometry was 220 nm.

In order to ensure the indentation of the nanoarray structures, a pressure of 10 MPa was applied to the stack of stamp/polycarbonate film/macroelectrode on a 25 × 25 mm^2^ for 10 min at 180 °C (release temperature of 80 °C). In order to remove any residual PC present in the holes, nanoarrays were cleaned by oxygen plasma for 4 seconds using an ICP-RIE system applying 4 mT pressure, 200 W coil power and 10 W platen power.

### 2.4. Surface Functionalization

NEAs were functionalized by dropping 10 μL of 10 μg mL^-1^ gliadin fragments (Abcam^®^) in 0.01 M phosphate buffer (pH 7.2) on the active area of the NEA and incubating for 2 h at 25 °C. Prior to the immobilization of the antigen on the polycarbonate surface, NEAs were dipped in a 5 M NaOH solution for 1 min and then rinsed with Milli-Q water.

The functionalized NEA was incubated with 10 μL of 10 μg mL^-1^ solution of anti-gliadin in 0.01 PBS (pH 7.2) at 25 °C for 60 min. The NEA was subsequently blocked with 1% BSA for 30 min and then the captured primary antibody was coupled with 10 μL of 10 μg mL^-1^ solution of goat anti-mouse IgG secondary antibody for 60 min at 25 °C.

All incubations were followed by thorough rinsing in buffer solution, and in all procedures, a wet filter paper was introduced in the incubation container to keep the environment humid.

Cyclic voltammograms were recorded between +0.2 and −0.6 V in a solution of 0.1 mM methylene blue in 0.01 M PBS. The mediator shuttles the electrons between the surface of each nanoelectrodes and the enzyme (HRP) at the end of the biomolecules chains resulting from the sequence biorecognition events and anchored to the dielectric surface surrounding the nanoelectrodes. HRP is reduced by the mediator and oxidized by H_2_O_2_ (Figure 1).

## 3. Results and Discussion

### 3.1. Characterization of NEAs

**Figure 2:**
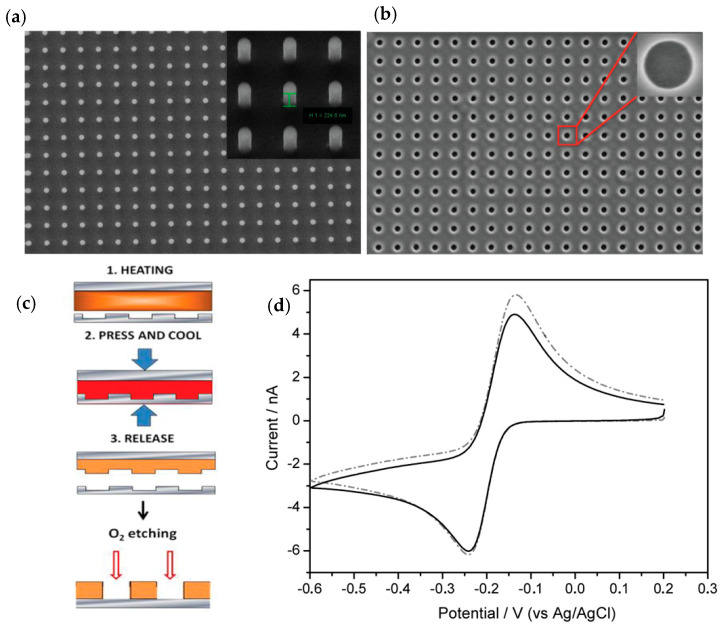
(**a**) SEM images of the stamp obtained for thermal nanoimprint lithography (TNIL) in front view and the inset in tilted view. Array of pillars have average diameter of 250 nm and interspacing distance of 800 nm and (**b**) SEM image of the NEA with dots spaced at the distance of 800 nm, an average diameter of ~260 nm and a depth of ~225 nm plus the magnification of one boron-doped diamond (BDD) nanoelectrode. (**c**) Schematic representation of the thermal nanoimprinting process. (**d**) Cyclic voltammograms of the bare NEA recorded in 0.1 mM MB in 0.01 M PBS at pH 7.2 (black line) with 50 µM of H_2_O_2_ (dashed line). Scan rate, 50 mV s^−1^.

NEAs were fabricated by TNIL process using a stamp consisting in a square array of nanopillars of 800 nm period, with diameter of 260 nm and a depth of 225 nm (Figure 2a). The stamp was used to indent NEAs on the polycarbonate film deposited on BDD electrode. After the imprinting, the samples were treated with oxygen plasma in order to remove the residual layer from the bottom of nanoelectrodes. Figure 2b shows a SEM image of the NEAs on BDD with dots with average diameter of 265 nm and interspacing distance of 800 nm. In Figure 2c, the TNIL process is represented.

NEAs were tested through cyclic voltammetry (CV) using methylene blue as reversible redox probe and 0.01 M PBS as supporting electrolyte.

**Figure 3:**
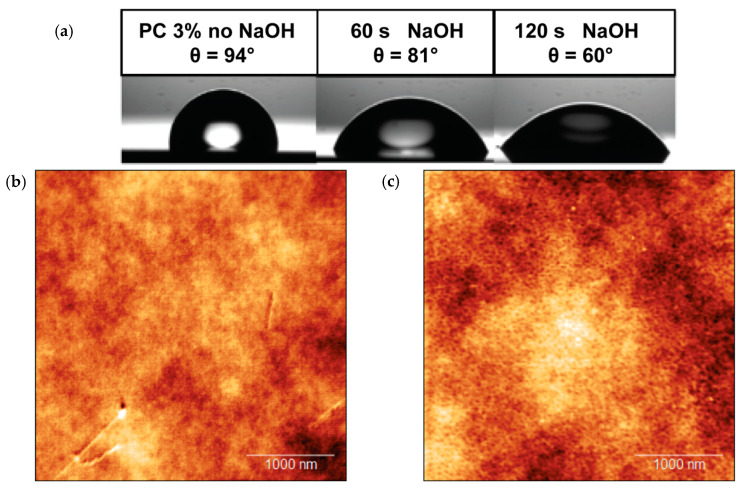
(**a**) Water contact angle measurement done on the bare PC surface and after the treatment with NaOH for 60 and 120 s. (**b**) Atomic force microscopy (AFM) images of the polycarbonate surface of the NEA before functionalization and (**c**) after gliadin adsorption.

Figure 3 shows a well-resolved reduction peak, which is attributed to the two electron–one proton reduction of MB to the leuco form (LB) [35]. These data demonstrate that the NEAs are electroactive and no residuals of PC, which can cause electrical resistance effects, are present on the surface of the electrodes. Moreover, it can be noticed that the cyclic voltammogram is peak-shaped, which is characteristic of linear diffusional regimes [15,20,27].

### 3.2. Detection of Gliadin Fragment on the Immunosensor Platform

To convert NEAs into a sensing platform, we took advantage of the possibility to immobilize specific molecules on the PC dielectric surface surrounding each nanoelectrode, rather than on the electrode itself. In this way, the electrochemical properties of the electrode and the physicochemical properties of the dielectric surface can be optimized independently facilitating the combination of highly specific molecular recognition mechanisms with high sensitivity and low detection limits [39,40].

As proof of this concept, we functionalized the PC surface with gliadin protein fragment (see Section 2 for details). Prior to functionalization, NEAs were dipped in a 5 M NaOH solution for 1 min and then rinsed with Milli-Q water. We found this step essential for the activation of the PC surface of NEAs and efficient functionalization with gliadin proteins. In fact, NaOH treatment leads to the deprotonation of hydroxyl and carboxylic groups naturally present on the PC surface, which acquires hydrophilic features. This phenomenon can give rise to electrostatic interactions that may be caused by a decreased lateral electrostatic repulsion between the proteins at the interface when the pH reaches the isoelectric interval [41], which for gliadins is between 6.5 and 8.1. The effect of this treatment on PC surface was investigated by contact angle measurement (Figure 3a).

In Figure 3, atomic force microscopy (AFM) images in air of the NEAs surface before (Figure 3b) and after (Figure 3c) the functionalization with gliadin fragments are presented. A difference in surface roughness (see Figure S1a,b) after functionalization can be appreciated. However, the surface presents an intrinsic roughness (≈4 nm) which hinders the detection of possible changes of topography due to gliadin physisorption.

After the NEAs functionalization, gliadin fragments were detected through a first incubation with the anti-gliadin-IgG primary antibody and subsequently with the specific secondary antibody anti-IgG labeled with horse radish peroxidase (HRP) as enzymatic probe (see Section 2 for details and Figure 1). Then, the originated electrochemical signal was detected through cyclic voltammetry (CV) in methylene blue (MB) after the addition of the substrate H_2_O_2_. We choose MB as redox mediator due to its well-known capacity to efficiently shuttle electrons from NEA to the HRP label [35].

**Figure 4:**
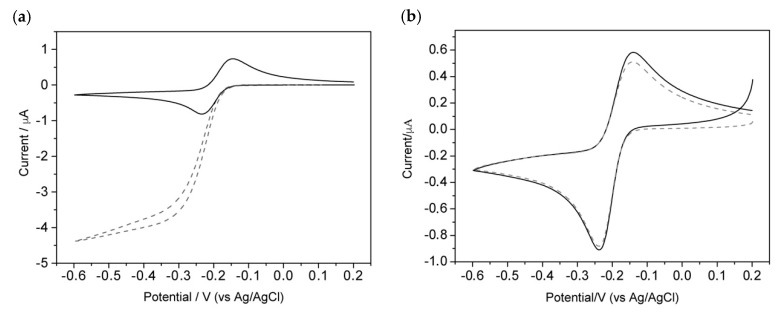
Cyclic voltammograms of (**a**) the immunosensor recorded in the absence (dashed line) and presence of 1.2 mM H_2_O_2_ and (**b**) the immunosensor without the incubation step with gliadin (negative control) recorded in the absence (dashed line) and presence of 1.2 mM H_2_O_2_. Scan rate, 50 mV s^−1^; supporting electrolyte, 0.1 mM MB in 0.01 M PBS; and pH 7.2.

When H_2_O_2_ is added to the treated NEA, a dramatic change in voltammetric pattern is detected: the reduction current increases and the oxidation peak disappears, with increasing concentration of H_2_O_2_ substrate (Figure 4a). It can be also noticed that the CV pattern becomes sigmoidal shaped in agreement with the occurrence of an electrocatalytic process, which involves the following reactions:

(1) $$\mathrm{MB}+2{\;e}^{-}+{\;H}^{+}\to \mathrm{LB}$$

(2) $${\mathrm{HRP}}_{\mathrm{red}}+H_{2}O_{2}+2H^{+}\to {\mathrm{HRP}}_{\mathrm{ox}}+2H_{2}O$$

(3) $${\mathrm{HRP}}_{\mathrm{ox}}+\mathrm{LB}\to {\mathrm{HRP}}_{\mathrm{red}}+\mathrm{MB}$$

This evident change in the voltammetric pattern also confirms the effective immobilization of gliadin on the PC of the NEA and subsequent reactions with primary and secondary antibodies.

To check for possible nonspecific adsorption of the primary and secondary antibodies on the PC surface, the procedure was repeated only after incubation with 1% BSA and after the addition of the primary and the secondary antibodies. From Figure 4b, it can be seen that no variation of the voltammetric response occur, excluding any nonspecific interaction between the secondary antigliadin-HRP and the NEA surface. All these evidences indicate that (i) gliadin fragments are captured by the NEA, (ii) gliadin reacts efficiently with the primary antibody anti-gliadin which successfully reacts with the secondary antibody anti-IgG labeled with HRP, thus gliadin is bound on the NEA surface, (iii) antigliadin-IgG and anti-IgG labeled with HRP do not bind on the electrode surface in the absence of gliadin, and (iv) the addition of H_2_O_2_ causes an electrocatalytic increase of the MB reduction current, which could be potentially employed for analytical purposes.

Furthermore, we did not detect any reduction of the current after the interaction of the NEA with gliadin and primary and secondary antibodies, thus indicating that the proteins are mainly bound to the PC surface instead of the BDD.

**Figure 5:**
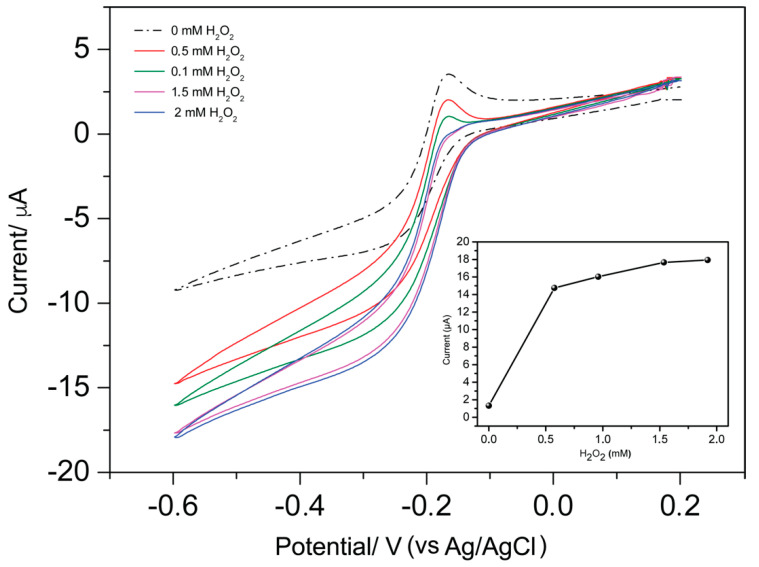
Cyclic voltammograms of the HRP-gliadin-NEA recorded in 0.1 mM MB in the presence of different concentrations of H_2_O_2_ (0–2 mM). Scan rate, 50 mV s^−1^; supporting electrolyte, 0.1 mM MB in 0.01 M PBS; and pH 7.2. Inset shows anodic peak current as function of H_2_O_2_ concentration.

To optimize the response of the so-prepared gliadin-NEA, the role of the concentration of the enzyme substrate H_2_O_2_ was assessed while keeping the concentration of gliadin constant at 1 µg mL^−1^. Figure 5 shows the changes in the CVs obtained with the HRP-gliadin-NEA for H_2_O_2_ concentrations in the range of 0–2 mM. The electrocatalytic reduction current shows an increase, whereas the oxidation peak shows decrease, with the hydrogen peroxide concentration up to 1.5 mM where it reaches a plateau.

**Figure 6:**
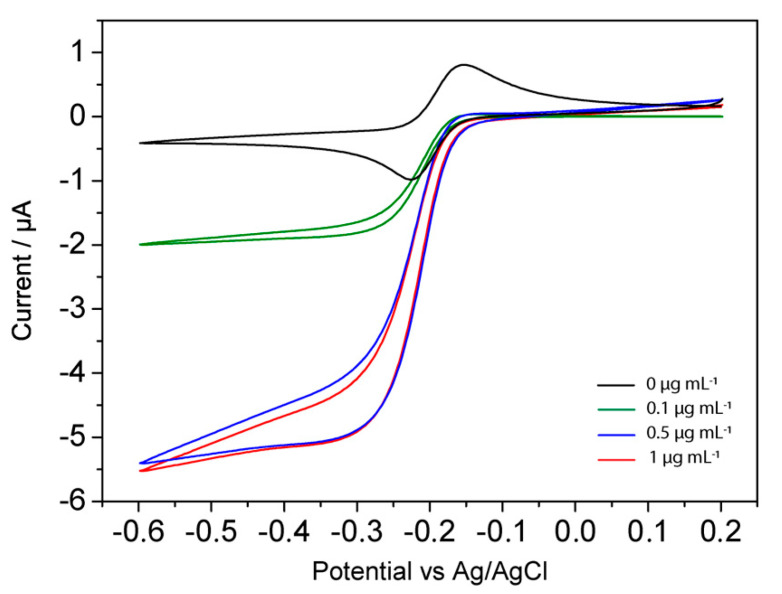
Cyclic voltammograms recorded with the NEA incubated with different concentrations of gliadin (0, 0.1, 0.5, and 1 µg mL^−1^) obtained in 0.1 mM MB in presence of 1.5 mM H_2_O_2_. Scan rate, 50 mV s^−1^; supporting electrolyte, 0.1 mM MB in 0.01 M PBS; and pH 7.2.

A series of experiments were performed incubating different NEAs with solution at different gliadin concentrations (0.5–10 μg mL^-1^). The CV responses of three representative concentrations are reported in Figure 6.

**Figure 7:**
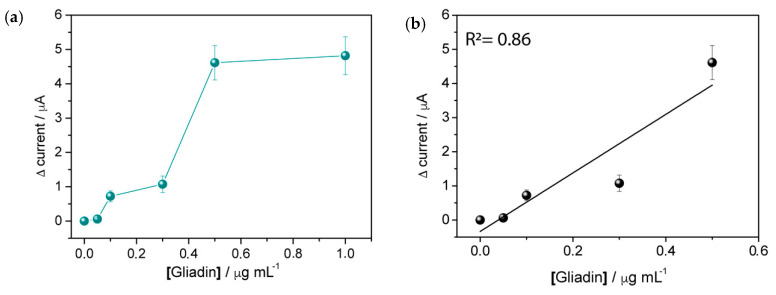
(**a**) Δ current as function of gliadin fragment concentration and (**b**) linear response for gliadin concentration ≤0.5 µg mL^−1^. Signal obtained by cyclic voltammograms in 0.1 mM MB in the absence and presence of decreasing concentrations of gliadin (0.5–10 μg/mL). Scan rate, 50 mV s^−1^; supporting electrolyte, 0.1 mM MB in 0.01 M PBS; and pH 7.2.

As shown in Figure 7a, the current values increase with increasing concentration of gliadin, tending to an asymptotic profile for concentrations greater than 0.5 µg mL^−1^ and suggesting that this is the maximum concentration of gliadin that we can immobilize on the PC surface using the method above described. Nevertheless, a linear trend (R^2^ = 0.86) can be identified for concentrations ≤0.5 µg mL^−1^ (see Figure 7b).

In order to study the stability of our platform during time, repeated detection of gliadin was performed after 1 and 7 days, by measuring the generated catalytic current (Figure S2). After each measurement, the sample was stored in 0.01 M PBS, pH 7.2, at 4 °C. The observed loss in sensitivity was 25% on Day 1 and 57% on Day 7 after the preparation of the sensor. These results suggest that the storage process does affect the enzymatic catalytic activity. However, given the simple protocol (see Section 2), functionalizing fresh NEA do not represent a main issue.

## 4. Conclusions

In the present study, we presented the fabrication by TNIL of an electrochemical sensor consisting in an array of nanoelectrodes by TNIL, by transferring arrays of nanoholes in a thin film of polycarbonate (PC) deposited on boron-doped diamond electrodes. Parameters for both the stamp optimization and TNIL patterning have been fine-tuned and electrochemical behavior of our NEAs has been tested.

Thanks to the efficient and rapid TNIL technology, the developed processes for the fabrication of NEAs have reached a stage in which they can be manufactured reliably and reproducibly. Therefore, this process might be object of further industrial development towards some market applications.

Furthermore, we demonstrated the possibility to efficiently immobilize biomolecules, on the relatively large surface of the PC of our NEAs, thus avoiding electrode passivation retaining all the advantages of NEA, such as high S/N ratio and higher sensitivity. Gliadin protein fragment detection was taken as a case study to demonstrate the general applicability of the concept. We demonstrated that gliadin fragments are captured by the NEA and reacts efficiently with the primary and secondary antibodies to form an immunocomplex labelled with HRP and thus electrochemically detectable. This method allows to detect and discriminate various concentrations of gliadin fragments showing a linear trend. Further optimizations regarding the surface functionalization will be required in order to standardize the method, to evaluate its clinical sensitivity and specificity, and to assess the matrix effect of this system.

To conclude, the creation of nanostructures on the sensing surface contributes to increasing the specific area available for the immobilization of high amounts of the biomolecules involved in the recognition event, while keeping the overall size of the sensor to very small figures. However, further optimizations are needed to define the detection limit of this system. Moreover, the characteristics of the TNIL technology in the fabrication of high-resolution structures down to the 10 nm scale and their low-cost production represent a good opportunity to scale up the production of NEAs-based electrochemical sensing platform to monitor biochemical molecules for both food ad biomedical applications.

## Figures and Tables

**Table 1:** Inductive coupled plasma (ICP) parameters used in the fabrication of the stamp: for the removal of residual layer after the thermal nanoimprint lithography (TNIL) process, for the pattern transfer into silicon, and for the ashing for the final resist removal.

	O_2_ Plasma	Fluorine-Based Plasma	Plasma Ashing
Coil power	200 W	400 W	800 W
Platen power	10 W	20 W	20 W
Flow	O_2_ 40 sccm	SF_6_ 30 sccm C_4_F_8_ 60 sccm Ar 10 sccm	O_2_ 50 sccm
Pressure	4 mT	8 mT	20 mT
BIAS	35 V	95 V	45 V
Time	15 s	15 s	15 s

## Supplementary Materials

The following are available online at https://www.mdpi.com/2079-6374/10/8/90/s1, Figure S1: AFM characterization, Figure S2: Gliadin-NEA performance during time.
