# Supplementary material for: Nanoelectrode Arrays Fabricated by Thermal Nanoimprint Lithography for Biosensing Application
Source: Biosensors (Basel). 2020 Aug 5;10(8):90. doi: 10.3390/bios10080090 (PMC7459808; doi:10.3390/bios10080090)
Supplement: Supplementary file 1 [file biosensors-10-00090-s001.pdf]

biosensors

MDPI

Supplementary Materials

Article

Nanoelectrode Arrays Fabricated by Thermal Nanoimprint Lithography for Biosensing Application

Alessandra Zanut [1,2,*,†], Alessandro Cian [3,4], Nicola Cefarin [1,2], Alessandro Pozzato [3] and Massimo Tormen [2,3]

1 Department of Physics, University of Trieste, P.le Europa 1, 34100 Trieste, Italy; cefarin@iom.cnr.it
2 IOM-CNR, TASC Laboratory, Area Science Park—Basovizza, S.S 14 Km 163.5, I-34149 Trieste, Italy; massimo.tormen@thundernil.com
3 ThunderNIL srl, via Foscolo 8, I-35131 Padova, Italy; acian@fbk.eu (A.C.); alessandro.pozzato@thundernil.com (A.P.)
4 Center for Materials and Microsystems, Fondazione Bruno Kessler, 38123 Trento, Italy
* Correspondence: az2218@nyu.edu
† Current address: Tandon School of Engineering, New York University, Brooklyn, NY 11201, USA.

Received: 01 July 2020; Accepted: 31 July 2020; Published: 4 August 2020

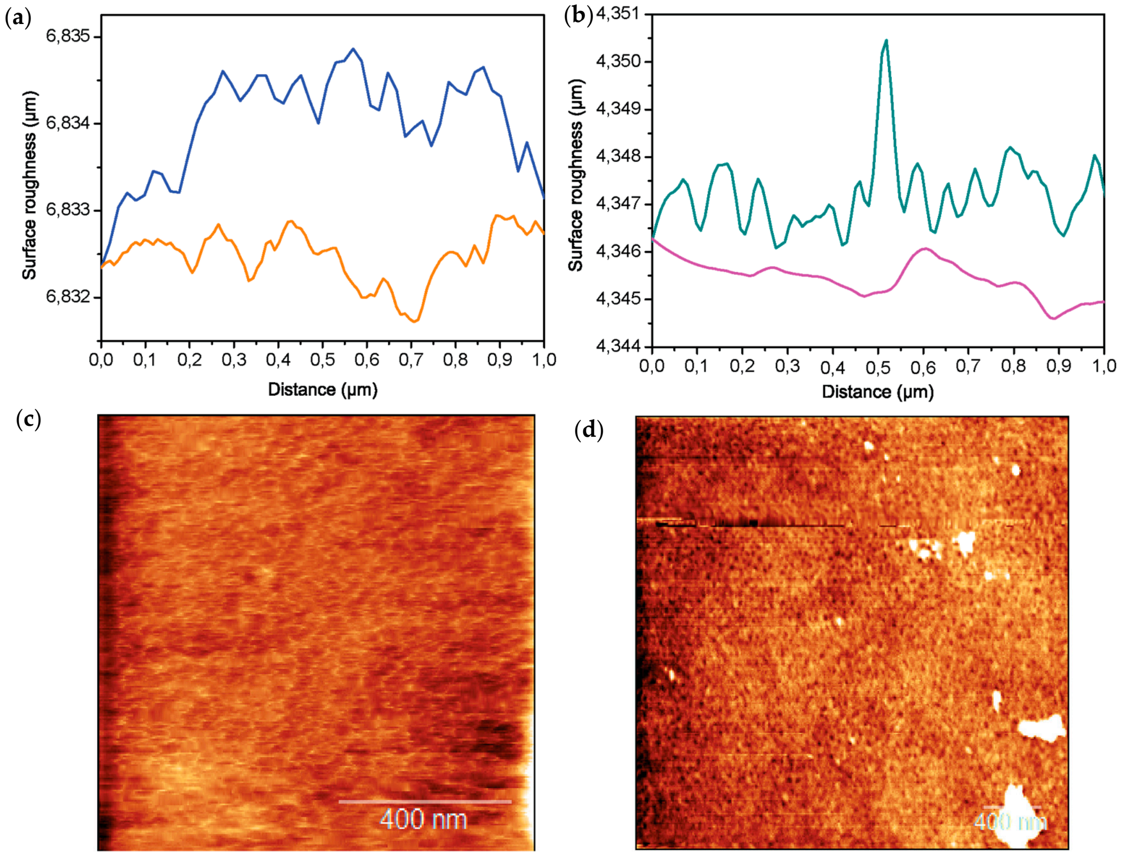

Figure S1. AFM characterization: (**a**) line profile of PC film deposited on BDD electrode reported in the main text in Figure 4a (orange line) and of PC film deposited on BDD and functionalized with gliadin fragments reported in the main text in Figure 4b (blue line); (**b**) line profile of (**c**) PC film deposited on silicon substrate (purple line) and of (**d**) PC film deposited on silicon substrate and functionalized with gliadin fragments (greenline).

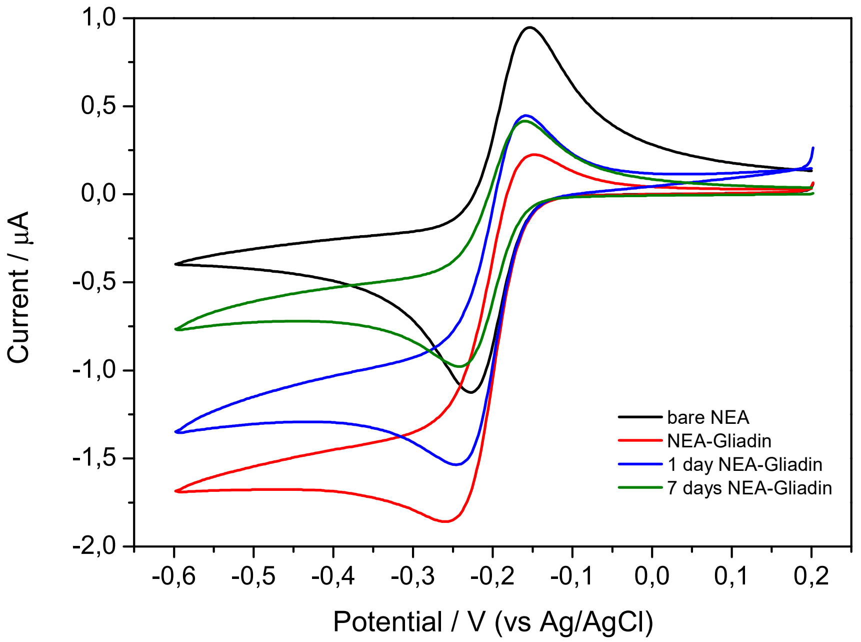

Figure S2. Gliadin-NEA performance during time. Cyclic voltammograms of the gliadin-NEA recorded in 0.1 mM MB in the presence of 1.2 mM H_2O_2 (red curve) after 1 day (blue curve) and after 7 days (green curve) from the functionalization. Scan rate, 50 mV/s; supporting electrolyte, 0.01 mM PBS; and pH, 7.2.
